# Instability of expanding bacterial droplets

**DOI:** 10.1038/s41467-018-03758-z

**Published:** 2018-04-03

**Authors:** Andrey Sokolov, Leonardo Dominguez Rubio, John F. Brady, Igor S. Aranson

**Affiliations:** 1 Materials Science Division, Argonne National Laboratory, Argonne, IL 60439 USA; 2 Department of Biomedical Engineering, Pennsylvania State University, University Park, PA 16802 USA; 3 Division of Chemistry and Chemical Engineering, California Institute of Technology, Pasadena, CA 91125 USA

## Abstract

Suspensions of motile bacteria or synthetic microswimmers, termed active matter, manifest a remarkable propensity for self-organization, and formation of large-scale coherent structures. Most active matter research deals with almost homogeneous in space systems and little is known about the dynamics of strongly heterogeneous active matter. Here we report on experimental and theoretical studies on the expansion of highly concentrated bacterial droplets into an ambient bacteria-free fluid. The droplet is formed beneath a rapidly rotating solid macroscopic particle inserted in the suspension. We observe vigorous instability of the droplet reminiscent of a violent explosion. The phenomenon is explained in terms of continuum first-principle theory based on the swim pressure concept. Our findings provide insights into the dynamics of active matter with strong density gradients and significantly expand the scope of experimental and analytic tools for control and manipulation of active systems.

## Abstract (web summary)

Sokolov et al. have previously shown how bacteria are expelled in response to a rotating microparticle. Here the authors find that when the microparticle is spun at much higher rotation rates bacteria are trapped around it and then are expelled radially upon rotation cessation in an explosion-like manner.

## Introduction

Collective motion and self-organization of motile suspensions is active topic of research^1,2^. Recent studies uncovered many salient features of active matter, both living^3–7^ and synthetic^8–10^. Various theoretical approaches, from discrete particle simulations^11,12^, probabilistic kinetic approaches^13,14^ to phenomenological hydrodynamic theories^15^ are used to describe various aspects of collective motion in active suspensions. It is appealing to use thermodynamic or state-like variables, e.g., pressure and temperature, in the context of active matter. In fact, recent studies demonstrated that while the thermodynamic analogy has limitations, many aspect of active suspension dynamics can be properly captured in the terms of swim pressure and corresponding equation of state^16–21^.

Most of the microswimmer research so far has focused on the dynamics of homogeneous active suspensions where the microswimmer density fluctuations are small^4,15,22^. In a related system of synthetic swimmers^8,9^, it was found that the self-propulsion can naturally cause some non-equilibrium densification and clustering termed motility-induced phase separation^23^. However, nothing is known about the dynamics of active matter with strong density heterogeneities. These conditions can be achieved, for example, by submersing a rapidly rotating solid macroscopic particle into bacterial suspension. The rotation leads to the expulsion of most bacteria from the particle but some bacteria is trapped near the particle^24^. The trapped bacteria are then released upon cessation of rotation.

In this study experiments are conducted at much higher rotation rates that lead to the formation of highly concentrated bacterial droplets with a range of concentrations not available in previous experiments. Most surprisingly, cessation of rotation leads to a violent explosion of the droplet. The observed behavior is reminiscent of the Richtmyer-Meshkov instability of two fluids of different density that are impulsively accelerated^25^. However, the analogy with the Richtmyer-Meshkov instability is somewhat deceiving. The instability of accelerating fluids is due to a volume force arising from the density difference. In contrast, for the bacterial droplets suspended in essentially zero Reynolds number fluid material acceleration is insignificant. Thus the instability is entirely due to self-propulsion and hydrodynamic interactions between the micro-swimmers. Here we characterize the observed instability experimentally using a variety of techniques, from fluorescent microscopy, particle-image velocimetry to optical coherence tomography. We establish that the droplet edge velocity increases with the droplet's local curvature, which provides a positive feedback mechanism driving the instability. Furthermore, we capture the onset of the instability in terms of a swim pressure concept generalized to rod-like particles such as bacteria. The model is derived in the limit when the Peclet number based on the swim diffusivity is small.

## Results

### Characterization of droplet instability

**Fig. 1:**
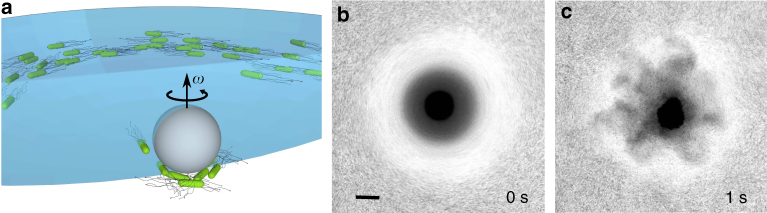
Illustration of droplet instability. **a** Three-dimensional schematic representation of the experimental setup. A 60 μm nickel particle is spun by an external magnetic field inside a pendant drop with swimming bacteria. Rotation of the particle creates a vortex; the vortex redistributes bacteria and forms a dense bacterial droplet. **b** Stable bacterial concentration distribution for rotation frequency of 400 Hz. Scale bar is 50 μm. **c** Vigorous explosion of the concentrated bacterial droplet 1 s after cessation of rotation

A pendant drop containing a bacterial suspension (about 8–10 μL) was attached to the bottom of a microscope glass slide. A magnetized nickel particle (30–40 μm radius) is placed in the droplet. Due to gravity, the particle sinks to the bottom of the drop and is kept at this position by capillary forces. The particle is spun with the frequency 100–400 Hz by a rotating horizontal magnetic field created by a pair of orthogonal Helmholtz coils, see Fig. 1a. Two main differences compared to our earlier experiment^24^ are: the rotation frequency was 10–20 times higher (100–400 Hz vs 2–20 Hz); the bacterial concentration was 100 times higher (2 × 10^10^ cm^−3^ vs 1–5 × 10^8^ cm^−3^ in ref.^24^).

These very different experimental conditions yield fundamentally new behavior, see Fig. 1b,c. In addition to the previously observed expulsion of bacteria from the particle (seen as a bright white halo in Fig. 1b), the high-frequency particle rotation (400 Hz) resulted in the formation of a highly compressed bacterial droplet beneath the particle seen as a dark accretion disk. We estimate that the concentration of bacteria in the droplet is about 10^11^ cm^−3^. Upon cessation of rotation, a violent instability of the disk is observed, reminiscent of an explosion, see Fig. 1c and Supplementary Movies 1 and 2. Reduction in the rotation frequency results in the reduction of the disk diameter, taming of the instability, and uniform expansion of the droplet, see Supplementary Movie 3. No instability was observed for the frequencies below 100 Hz.

### Fluorescent and tomography experiments

**Fig. 2:**
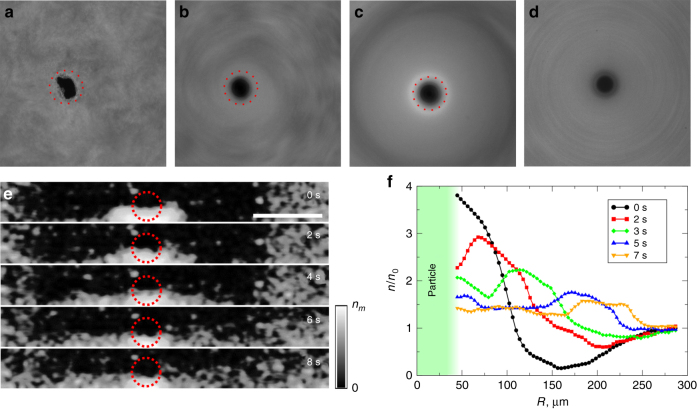
Fluorescence and tomography analysis. **a**–**d** Images of fluorescent bacteria in the vicinity of a rotating particle. White color corresponds to higher concentration of bacteria. Images are made from the bottom. The frequency of rotation is 160 Hz. **a** The initial distribution of live bacteria near the bottom of the film before the onset of rotation. **b** Uniform distribution of bacteria immediately after the onset of rotation. **c** Stationary distribution of swimming bacteria around the rotating particle. Bacteria are concentrated in the close proximity of the particle. Red dashed circles illustrate the size of the particle. **d** Distribution of dead (non-motile) bacteria remains uniform. Scale bar is 50 μm. **e** The OCT images showing bacterial distribution around the particle in a vertical cross-section at different moments of time after cessation of rotation. The OCT probe is scanning from the bottom. Bright white color corresponds to higher bacterial concentration, *n*_*m*_ = 10^11^ cm^−3^. The bottom part of the spinning particle (dashed red circle) and concentrated bacteria are visible as a bright spot near the center of the first image. The droplet of concentrated bacteria is expanding along the bottom surface of the film in four consecutive images. Scale bar is 200 μm. **f** Averaged radial distributions of bacteria around the particle at different moments of time after cessation of rotation. *n*_0_ is concentration of bacteria far from the particle

In order to characterize the distribution of bacteria near the surface, we conducted experiments with fluorescent bacteria, see Fig. 2a–d. Two different fluorescent strains, DK3394 (green) and DK400 (red), of swimming bacteria *Bacillus subtilis*, were used. Strain DK400 was killed before the experiment and then mixed with live strain DK 3394. From the fluorescence intensities of green and red colors we established the densities of active swimmers and non-motile (dead) bacteria. The non-motile bacteria have an advantage compared to passive tracers: they have the same size, shape, and density as the living bacteria, and therefore are affected by the centrifugal forces in the same fashion. From the fluorescent images, we established that the motile bacteria are concentrated under the spinning particle, Fig. 2c. In contrast, non-motile bacteria are uniformly distributed, Fig. 2d.

We also characterized the three dimensional distribution of bacteria in the vicinity of spinning particle by optical density measurements. For this purpose we used optical coherence tomography (OCT), a non-invasive low coherence interferometry technique that uses infrared light to measure optical scattering profile of the media. From the OCT images, Fig. 2e, we established that the bacteria are depleted in the bulk of the fluid droplet and accumulated under the particle in the thin accretion disk. We can also see from these images that upon cessation of rotation, the bacteria expand in a relatively narrow layer near the open surface of the droplet, Fig. 2e and Supplementary Movie 4. The height of the expanding layer is comparable with 1/2 particle diameter or 5–6 bacterial lengths. The height of the dense layer remains roughly the same during the expansion, while the horizontal size changes from 2–3 particle diameters (20 bacterial lengths) to 5–6 particle diameters (60 bacterial lengths), as can be seen in Fig. 2e. In addition, we did not see in the experiments strong density modulations within the expanding layer in the vertical direction. Fig. 2f illustrates evolution of bacterial radial distribution. The overall diffusive-like behavior is qualitatively similar to that observed in early experiments on acoustic trapping of microswimmers^17^.

Thus, both fluorescence and optical tomography measurements prove that accumulation of bacteria occurs in a relatively thin and dense accretion disc located under the particle. These observations justify the quasi-two-dimensional description of the phenomenon.

### Instability of the droplet interface

**Fig. 3:**
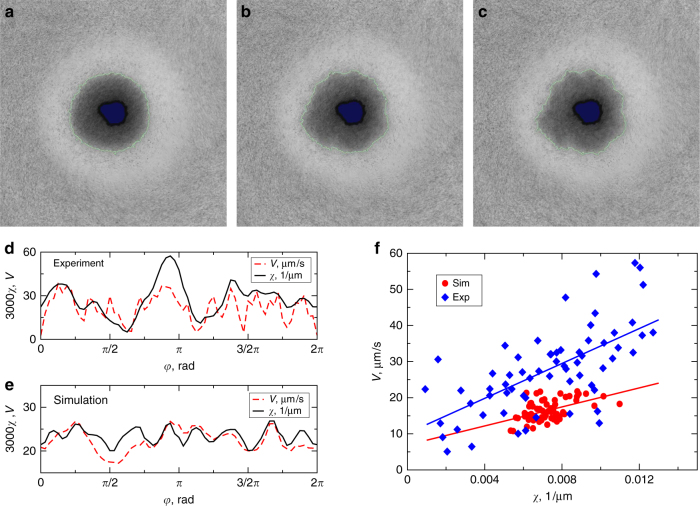
Droplet's interface instability. The sequence of three consecutive snapshots illustrating the evolution of the dense droplet for times *t* = 0.15 s (**a**), *t* = 0.3 s (**b**), and *t* = 0.4 s (**c**) after cessation of rotation. The interface between dilute (bright) and dense (dark) regions is shown in green line. Rotation frequency is 400 Hz. Dependence of the interface velocity *V* (green) on the interface curvature (*χ*) for the exploding droplet 0.2 s after cessation of rotation. **d**, **e** Dependence of the interface velocity *V* (dashed red) and the interface curvature *χ* (solid black) on the polar angle *φ* for the experimental data (**d**) and the data obtained from simulations (**e**). A noticeable correlation between *V* and *χ* is observed both in experiments and simulations. **f** Parametric dependence of the interface normal velocity *V* vs curvature *χ* for experimental data (blue diamonds) and simulations (red circles). Two lines are linear regression fits

From the experimental images and results of computational simulations discussed below, we tracked the interface between dense and dilute regions after cession of rotation. From the position of the interface in consecutive frames, we calculated the normal interface velocity *V* and its local curvature *χ*. The results are shown in Fig. 3. Just after cession of rotation, the edge (interface) of the dense bacteria droplet was tracked. The droplet's interface was divided into 64 angular sectors with respect to the center of rotation, see Fig. 3a–c. The local curvature *χ* and interface normal velocity *V* were calculated for each frame in the experiment Fig. 3d and simulation Fig. 3e. Despite the experimental noise, one can see the obvious correlation between *V* and *χ*. Overall, the velocity increases with the curvature; see Fig. 3f, consistent with a linear law

1 $$V = V_0 + D_0\chi {\kern 1pt} ,$$

where linear regression gives the following values: *V*_0_ = 10 μm s^−1^, *D*_0_ = 2400 μm^2^ s^−1^ for experiments and *V*_0_ = 7 μm s^−1^, *D*_0_ = 1400 μm^2^ s^−1^ for simulations. This behavior is a hallmark of a long-wave instability of the interface. The instability characteristic length practically does not depend on the rotation frequency and is roughly 50–100 microns.

## Discussion

**Fig. 4:**
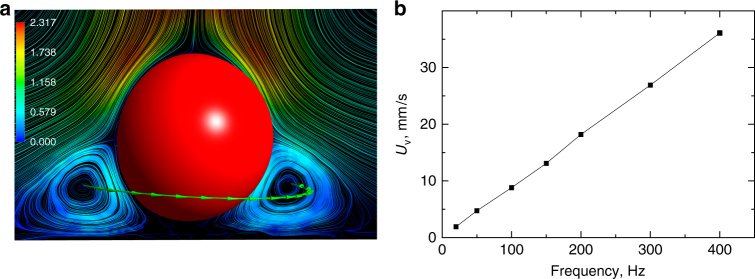
Stagnation zone. **a** A stagnation zone formed by a sphere rotating around an axis perpendicular to a plane: stream surfaces in a plane containing the rotation axis for *f* = 400 Hz, radius of the particle *a* = 30 μm. Velocity is measured in mm s^−1^. **b** Magnitude of the vortex velocity *U*_v_ vs frequency

The underlying reason for the concentration of bacteria under a rotating particle is a stagnation zone due to the proximity of a boundary^26^. The computed flow distribution for typical experimental conditions (frequency of rotation *f* = 400 Hz, particle radius 30 μm) is shown in Fig. 4a. The formation of a stagnation zone is a finite Reynolds number effect due to fluid inertia. We observe that swimming bacteria are accumulated in the stagnation zone. The computational analysis shows that the size of the stagnation zone is practically independent of the rotation frequency. However, the circulation flow magnitude increases linearly with the rotation frequency, Fig. 4b.

In the Stokes limit (zero Reynolds number Re), a sphere rotating in an unbounded fluid around a vertical axis produces pure azimuthal flow with velocity *V*_*φ*_ = *ωR*^2^ cos(*θ*)/*r*^2^, where *R* is the sphere radius, *r* distance from the center of rotation, and *φ*, *θ* are the corresponding azimuthal and polar angles. Due to fluid inertia (Re > 0), the centrifugal effect produces a swirling radial outflow in the equatorial region of the sphere. The radial flow must be maintained by a flow towards the poles along the axis of rotation. A boundary slows down the poleward flow in its vicinity, which is therefore no longer balanced by the poleward flow from the opposite side. As a result, the radial flow is directed towards the plane. This causes the formation of a stagnation ring^26^. Consequently, some fluid is trapped between the radial jet and the plane. As we have shown in^24^, the combined effect of the bacteria motility and curvature of the flow streamlines leads to migration of bacteria across the streamlines and accumulation in the stagnation zone. An increase in the rotation frequency increases the flow strength, and, consequently, results in a larger number of trapped bacteria.

**Fig. 5:**
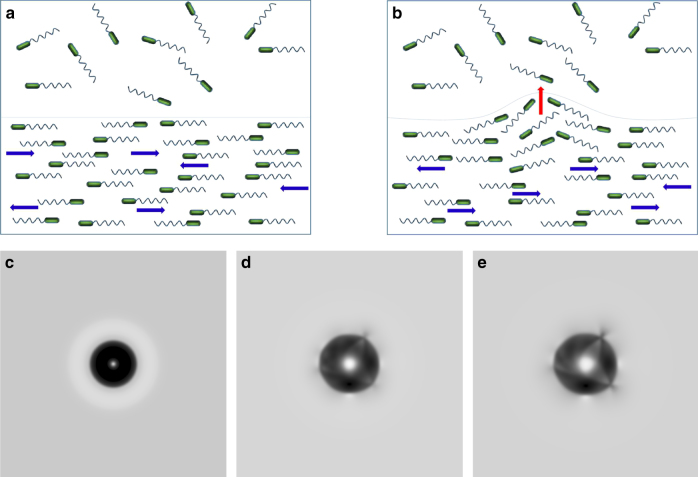
Expansion of a spot. **a**, **b** The onset of polar order (indicated by red arrow) from a slightly perturbed nematic state. **c**–**e** A sequence of gray-scale images illustrating expansion and instability of the dense spot for *t* = 2 (**c**), *t* = 25 (**d**), *t* = 34 (**e**) dimensionless time units after cessation of rotation. The nondimensional concentration field *n* is coded from white (*n* = 0) to black (max(*n*)). Initial spot radius is *r*_0_ = 20, and initial nondimensional concentration is *n* = *n*_0_ = 8. Outside the spot, the concentration is *n* = *n*_1_ = 0.85, i.e., dimensional *n* < *n*_c_. A depletion zone, seen as a bright halo, is imposed by initial conditions *n* = 0.2 in the domain *r*_0_ < *r* < 2*r*_0_, see panel (**c**). For the parameters of computational model see Methods section

Eqs (5) and (6) governing the evolution of the bacterial concentration *n* and the nematic order parameter **Q** (see Methods section) are one of the simplest models of an active nematic^27,28^. This model exhibits large-scale spatio-temporal chaos represented by random-like break-up, drift, and re-connection of nematic band-like solutions. It was shown in ref.^27^ that planar stationary nematic bands are always unstable in a large domain. The underlying reason is the generation of polar order from a slightly deformed nematically aligned state. Imagine a planar interface separating an isotropic state from a nematically ordered state of self-propelled particles moving on opposite tracks parallel to the interface, see Fig. 5a. A small deformation of the interface leads to the onset of local polar order and streaming of self-propelled particles perpendicular to the interface into the adjacent isotropic region, and, ultimately, amplification of the initial deformation, Fig. 5b. Simple calculations (see Methods section) yield that distortion of the nematic order (induced polarization) **P** = −*U*_0_*τ*_R_∇ ⋅ **Q**, where *U*_0_ is the swimming speed of the bacteria and *τ*_R_ is their reorientation or tumbling time, and their product is the run length $\ell = U_{0}\tau _{\rm R}$. Furthermore, a generalization of the band instability to the case of a planar front yields the following linear approximation of the relation between the normal front velocity *V* and its local curvature *χ*: *V* = 4*D*^swim^*χ*, where $D^{{\mathrm{swim}}} = U_0^2\tau _{\mathrm{R}}{\mathrm{/}}2$ is the swim diffusivity in 2D. This expression qualitatively agrees with the experimental value of *D*_0_ ≈ 2400 μm^2^ s^−1^ obtained for the configuration shown in Fig. 3d,f. The significant scattering of experimental data can be attributed to such factors as initial noise in bacterial length, orientation, swimming speed and image processing errors. While the linear dependence between the front velocity and the local curvature was clearly present in every experiment, we observed noticeable fluctuations between the experiments due to an uncontrollable variability of the initial conditions. Therefore, we avoided averaging results over many different experiments.

However, the experimental situation does not correspond to a stationary band but to an expanding spot. Therefore, as an initial condition, we set a localized circular spot of a radius *r*_0_. Inside the spot, we set $n = n_0 \gg n_{\mathrm{c}}$, $\left| {\bf{Q}} \right| = Q_0$, i.e., the homogeneous stable nematic state; here, *n*_c_ is the critical concentration for the isotropic to nematic transition. Outside the spot *n* = *n*_1_ < *n*_c_, **Q** = 0, i.e., the isotropic state. In additions, a depletion zone with *n* < *n*_1_ was imposed by initial conditions in the domain *r*_0_ < *r* < 2*r*_0_ to model more closely the experimental situation. Inside the spot the orientation of the nematic was chosen parallel to the spot boundary. This assumption is consistent with our observation^24^ that a vortical flow aligns the bacteria along the streamlines. The nematic state is then stabilized by the rotation. Cessation of rotation triggers the onset of the instability. The depletion zone in our simulation was introduced for better correspondence to the experiment. As a matter of fact, the instability of the droplet occurs even without a depletion zone as long as the density outside the droplet remains below the nematic threshold, although with somewhat smaller growth rate.

The computational results can be summarized as following, see Fig. 5 and Supplementary Movie 5. As in the experiment, the spot initially expands due to the high internal swim pressure. If the initial bacterial concentration was not too large, then the spot expanded smoothly and no instability was observed. In the experiment, this situation corresponds to slow rotation rates,  *f* < 100 Hz. However, for a large enough initial concentration the expansion becomes unstable and explosion-like, see Fig. 5, as it was seen for higher rotation rates, compare Fig. 1c. While the detailed shapes of the exploding droplets show more angular variation than our simplified model, the computational modeling qualitatively agrees with the experiments. Furthermore, computational model and the experiment show similar correlation between interface velocity and local curvature, compare Fig. 3d,e. For the permissible values of swim diffusivity, the slope of the computational dependence of *V* vs *χ* in Fig. 3f is lower than the experimental one. This is likely due to model assumption: we neglected advection of bacteria by the fluid, which possibly would increase the droplet expansion rate.

In conclusion, we have described a generic instability of a highly compressed bacterial droplet. The observed instability is interpreted in terms of a simple model derived from conservation laws for the microswimmer local concentration and linear momenta. The model yields qualitative agreement with the experiments. Further refinements, such as taking into account large-scale flow generation, may result in an even better agreement.

Depletion of bacterial concentration by a rotating particle in a dilute regime was a focus of our previous study^24^. Accumulation of bacteria was observed as well, but the nature of the accumulation was not clarified. More importantly, no instability was observed. As the theoretical description is concerned, in a certain limit Eqs. (5) and (6) are similar to that of refs.^27,29^. However, our starting point is very different. In contrast to^27,29^, there is an additional source of an anisotropic stress (swim pressure). The notion of swim pressure was needed to estimate the model parameters. The observed behavior cannot be interpreted in terms of a generic instability of a homogeneous nematic state^30^ near the onset of nematic-isotropic transition. In contrast, our instability occurs in strongly heterogeneous high density systems whereas the homogeneous nematic state is stable.

While the fluid flow formally can be neglected in the limit of small Peclet number^16^, the fluid motion probably does play a role, especially after the onset of the instability when the radial flow becomes comparable with the bacteria swimming speed. However, as a first modeling framework, we can use the simplest model to explain the experimental observations. The underlying reason for the instability is the onset of polar order from a slightly deformed nematic state. This instability mechanism is generic and not system-specific. Thus, we anticipate that our results may have relevance for a broad class of active systems under extreme conditions, such as microtubule gliding assays^31^, realizations of active nematics^6,32^, and even for a variety of synthetic active matter systems^8,33^.

## Methods

### Bacterial strains preparation

Three different strains of *Bacillus subtilis* were used in our experiments. For bright field microscopy and OCT measurements we used strains 1085 inoculated on a LB (Lysogeny broth) agar plate and then grown in Terrific Broth growth medium at 30 °C. The bacteria were extracted from the growth medium by centrifugation at the end of their exponential growth stage and washed. For fluorescent microscopy, we used strains DK400 (mCherry) and DK3394 (mNeonGreen) grown in Terrific Broth with the addition of isopropyl *β*-D-1-thiogalactopyranoside (IPTG, Sigma Aldrich) at 1 mM concentration. Bacteria DK400 were killed by transferring them to a glass container and heating to 90 °C, and then cooling to room temperature. Then dead bacteria DK400 were mixed with live bacteria DK3394 at the same ratio. The desired concentration in the range of 2 × 10^10^ cm^−3^ was made by additional centrifugation. Concentrated bacteria were transferred to a microscope glass plate with a prepared nickel particle by a digital micropipette. With the exception of the OCT experiments, the drop was enclosed in a small optically clear chamber 7 mm × 7 mm by 2 mm thick to minimize evaporation. OCT scanning requires placing an optical probe at a distance of a fraction of millimeter from the bottom of the drop. Scanning time for each frame is ~1 s.

### Equations of motion

The observed phenomena can be interpreted in the framework of the swim stress and from the conservation laws for the microswimmers' number density *n* and linear momenta^16,17,20,34^. The mixture of bacteria and fluid forms a suspension, which we model as a continuum with stress tensor ***σ*** and suspension velocity *u*. Both quantities are averages over a volume element containing both bacteria and fluid. The suspension velocity is incompressible, ∇ ⋅ **u** = 0, and the momentum balance for the suspension is *ρD***u**/*Dt* = ∇ ⋅ ***σ***, with *D*/*Dt* being the material derivative. The constitutive equation for the suspension stress is ***σ*** = ***σ***_f_ + ***σ***_p_, where ***σ*** = −*p*_f_**I** + 2*η***e**, is the usual stress tensor for a fluid, with *p*_f_ the pressure in the fluid and **e** the rate of strain tensor.

The particle contribution to the suspension stress, ***σ***_p_, arises from the activity of the swimmers, and takes the following form^16,34^

2 $${\boldsymbol{\sigma }}_{\mathrm{p}} = - \zeta D^{{\mathrm{tr}}}{\kern 1pt} n{\bf{I}} - \zeta D^{{\mathrm{swim}}}\left( {n{\bf{I}} + 2{\bf{Q}}} \right),$$

where *D*^tr^ is the translation diffusivity giving the effective osmotic pressure, $D^{{\mathrm{swim}}} = U_0^2\tau _{\mathrm{R}}{\mathrm{/}}2$ is the random-walk swim diffusivity (in 2D) due to activity, **I** is the identity tensor, *ζ* is the viscous drag coefficient of the swimmers, and **Q** is the nematic order tensor describing the orientation of the bacteria, which is a symmetric traceless 2 × 2 tensor. The translational diffusion *D*^tr^ = *D*^T^ + *D*^tum^ includes thermal diffusivity, *D*^T^ = *k*_B_*T/ζ*, *k*_B_ is the Boltzmann constant, *T* is the temperature. Translational diffusion *D*^tum^ arises due to displacements of the bacterium center of mass in the course of random tumbling event. This center of mass displacement is due to non-uniform distribution of flagella over the bacterial body: when the bacterium tumbles, it unbundles the flagella^35^. The forces from flaggelar motors applied at random positions of the bacterial body results in random reorientation of the body and random displacement of the center of mass. In most situations, $D^{\mathrm{T}} \ll D^{{\mathrm{tum}}}$ and $D^{{\mathrm{swim}}} \gg D^{{\mathrm{tum}}}$.

For the geometry of the problem we model the swimming as two dimensional. The diffusivity *D*^tr^ acts to stabilize the system at small wavelengths. Eq. (2) is the proper generalization of the swim pressure to its full tensorial form^34^. The swim stress is defined as the moment of the swim force ${\boldsymbol{\sigma }}_{\mathrm{p}} \equiv - n\left\langle {{\bf{xF}}^{{\mathrm{swim}}}} \right\rangle$, with **F**^swim^ = *ζU*_0_**q**(*t*), where *q*(*t*) is the orientation of the bacteria, and ${\bf{x}} = {\int}_0^t {\kern 1pt} {\bf{U}}\left( {t{\prime}} \right){\rm d}t{\prime}$. From the overdamped Langevin equation for the bacterial motion it follows that ${\boldsymbol{\sigma }}_{\mathrm{p}}$ = −$n\zeta U_0^2{\int}_0^t {\kern 1pt} \left\langle {{\bf{q}}(t){\bf{q}}(t{\prime})} \right\rangle {\rm d}t{\prime}$ = −$\zeta U_0^2\tau _{\mathrm{R}}{\kern 1pt} n{\bf{qq}}$. Using the identity $n{\bf{qq}} = {\bf{Q}} + \frac{n}{2}{\mathrm{tr}}({\bf{qq}}){\bf{I}}$, where ${\bf{Q}} = n{\bf{qq}} - \frac{n}{2}{\mathrm{tr}}({\bf{qq}}){\bf{I}}$ is traceless symmetric tensor, we obtain expression (2).

A local number density (or concentration) *n* obeys the usual conservation law

3 $$\partial _tn + \nabla \cdot {\bf{j}}_{\mathrm{p}} = 0,$$

where the particle flux **j**_p_ can be obtained either form a linear moment balance of active particles treated as a phase in the mixture of particles and fluid: −*ζ***j**_p_ + ∇ ⋅ ***σ***_p_ = 0, or from the Smoluchowski equation in position and orientation space for the probability density for swimmers. Both approaches give the same result:

4 $$\partial _tn + \nabla \cdot \left( {\frac{1}{\zeta }\nabla \cdot {\boldsymbol{\sigma }}_{\mathrm{p}}} \right) = 0.$$

In writing Eq. (4) we have neglected any advection of the swimmers due to the fluid suspension motion, which requires the Peclet number based on the swim diffusivity to be small. For small Peclet numbers, which we discuss below, the motion of the swimmers can be decoupled from that of the suspension and therefore we do not need to find the suspension velocity field. Also, for simplicity we have neglected any 'collisional' contributions to the stress and thus Eq. (2) contains the leading order effects of the bacterial concentration. The evolution of *n* is then given by

5 $$\partial _tn = D^{{\mathrm{eff}}}\nabla ^2n + 2D^{{\mathrm{swim}}}\nabla \nabla :{\bf{Q}}.$$

where *D*^eff^ = *D*^tr^ + *D*^swim^. The evolution equation for the nematic order tensor follows from the Smoluchowski equation in position and orientation space for the probability density for swimmers; its simplest form in 2D can be written as^36^

6 $$\partial _t{\bf{Q}} = - \xi \left( {1 - n{\mathrm{/}}n_{\mathrm{c}}} \right){\bf{Q}} - \mu \left| {\bf{Q}} \right|^2{\bf{Q}} + D^{{\mathrm{eff}}}\nabla ^2{\bf{Q}} + D^{{\mathrm{swim}}}\left( {\nabla \nabla - \frac{1}{2}{\bf{I}}\nabla ^2} \right)n.$$

Here, *ξ*(1 − *n*/*n*_c_)**Q** describes the onset of nematic order above the critical concentration *n*_c_, and the nonlinear term $\left| {\bf{Q}} \right|^2{\bf{Q}}$ is needed for saturation of the exponential growth; $\left| {\bf{Q}} \right|^2 = {\bf{Q}}:{\bf{Q}}$. From the Smoluchowski equation for nematics^36^, *ξ* = 2/*τ*_R_ and *μ* = 2*ξ*/*n*^2^. Experimentally, the rotational diffusion (reorientation) was *D*_R_ ≈ 1/*τ*_R_ = 0.05–0.1 rad^2^ s^−1^, and the average bacterial swim speed *U*_0_ ≈ 10–15 μm s^−1^, which gives a swim diffusivity $D^{{\mathrm{swim}}} = U_0^2\tau _{\mathrm{R}}{\mathrm{/}}2 \approx 500 - 2250$ μm^2^ s^−1^. For the thermal diffusivity we take *D*^T^ ≈ 0.05 μm^2^ s^−1^. According to ref.^24^, the value of translational diffusion *D*^tr^ ≈ 20–70 μm^2^ s^−1^, i.e., $D^{{\mathrm{tr}}} \gg D^{\mathrm{T}}$. The run length, $\ell$ = *U*_0_*τ*_R_ ≈ 70–100 μm, likely determines the characteristic length of the instability.

Writing Eqs. (5) and (6) in this continuum perspective implies that the polar order field, *m*, is slaved to the concentration and nematic fields. The polar order field evolution can be derived from the governing Smoluchowski equation, yielding

7 $$\partial _t{\bf{m}} + \nabla \cdot {\bf{j}}_{\mathrm{m}} + {\bf{m}}{\mathrm{/}}\tau _{\mathrm{R}} = 0,$$

where the polarization flux **j**_m_ = −*D*^tr^∇**m** + *U*_0_**Q** + *U*_0_*n***I**/2 (see ref.^20^ for details). The resulting equations were investigated in ref.^29^. They exhibit overall similar behavior to the much simpler Eqs. (5) and (6), including spatio-temporal chaos and band instability. Neglecting the time derivative and the Laplacian of **m** in Eq. (7), the polarization can become slaved to the concentration and nematic fields: **m** = −(*U*_0_*τ*_R_/2)∇*n* − *U*_0_*τ*_R_∇ ⋅ **Q**. Substituting this into the corresponding equations for the concentration and nematic fields yields Eqs. (5) and (6).

Finally, for the Peclet number, Pe = *U*^flow^*l*/*D*^swim^, we take the characteristic length scale the bacterial size, *l* ≈ 5 μm, the swim diffusivity *D*^swim^ ≈ 10^3^ μm s^−1^ and the observed flow velocity *U*^flow^ ≈ 50 μm s^−1^, which gives Pe ≈ 0.25, justifying the neglect of fluid advection on the concentration and nematic fields.

Here we emphasize that the concept of swim pressure is distinct from the active particle stress arising from the hydrodynamic stresslet for non-spherical particles, see e.g., refs.^11,13^. The ratio of the magnitude of the hydrodynamic stresslet to the swim stress is proportional to the ratio of the bacteria size to the run length^16^, $l{\mathrm{/}}\ell$ = *l*/(*U*_0_*τ*_R_) < 0.1 in these experiments.

### Instability of planar front

Stability of a stationary nematic planar band solution in the framework Eqs. (5) and (6) were studied in^27,29^. It was proved that the nematic band is always unstable with respect to long-wave undulations. Here we present a simplified analysis for a planar front. The result can be deduced from the instability of a band^27^ when the band's width diverges.

Consider 1D stationary front solution to Eqs. (5) and (6): *n* = *n*_0_(*y*), *Q*_*xx*_ = *Q*_0_(*y*), *Q*_*xy*_ = 0. Functions *n*_0_, *Q*_0_ satisfy equations

8 $$D^{{\mathrm{eff}}}\partial _y^2n_0 - 2D^{{\mathrm{swim}}}\partial _y^2Q_0 = 0$$

9 $$\xi \left( {n_0{\mathrm{/}}n_{\mathrm{c}} - 1} \right)Q_0 - 2\mu Q_0^3 - \frac{{D^{{\mathrm{swim}}}}}{2}\partial _y^2n_0 + {D^{{\mathrm{eff}}}}\partial _y^2Q_0 = 0$$

Here we denoted *D*^eff^ = *D*^swim^ + *D*^tr^. Concentration *n*_0_ can be expressed from the first equation

10 $$n_0 = \frac{{2D^{{\mathrm{swim}}}}}{{D^{{\mathrm{eff}}}}}Q_0 + \bar n$$

where $\bar n$ = *n*_0_(*y* → −∞) = const. Plugging *n*_0_ into the second equation, we obtain an equation for *Q*_0_

11 $$\xi \left( {n_0{\mathrm{/}}n_{{\mathrm{c}}r} - 1} \right)Q_0 -2 \mu Q_0^3 + {{\tilde D}}\partial _y^2Q_0 = 0$$

where $\tilde D = D^{{\mathrm{eff}}} - \frac{{\left( {D^{{\mathrm{swim}}}} \right)^2}}{{D^{{\mathrm{eff}}}}}$. Some exact solutions in the form of a stationary front or nematic band are obtained in^27,29^.

Here we examine front stability with respect to transverse undulations by considering solution in the form

12 $$n \approx n_0(y + y_0(x,t))$$

13 $$Q_{xx} \approx Q_0(y + y_0(x,t)) $$

14 $$Q_{xy} \approx P_0(y + y_0(x,t))$$

We assume that the front position is slowly changing in time *t* and along *x*, i.e., $\partial _xy_0 = O(\epsilon ) \ll 1$. Here *P*_0_ ~ *O*($\epsilon$) is a polarization induced by the front deformations.

Substituting Eqs. (12), (13) and (14) into Eq. (6), we obtain in the lowest order in *ε* (linear in *P*_0_)

15 $$\xi \left( {n_0{\mathrm{/}}n_{\mathrm{c}} - 1} \right)P_0 -2 \mu Q_0^2P_0 + {D^{{\mathrm{swim}}}}\partial _xy_0\partial _y^2n_0 + {D^{{\mathrm{eff}}}}\partial _y^2P_0 = 0$$

Here we neglected the higher-order terms like (∂_*t*_*x*_0_)∂_*y*_*P*_0_,(∂_*x*_*y*_0_)^2^∂_*y*_*P*_0_ etc. Using Eq. (11), we immediately see that it is satisfied if we set

16 $$P_0 = - 2\partial _xy_0Q_0$$

As we can see from Eq. (16), a front deformation (∂_*x*_*y*_0_ ≠ 0) results in the onset of polarization. Now, substituting Eq. (16), in Eq. (5), we obtain in the leading order (using Eq. (10))

17 $$\begin{array}{l}\partial _ty_0\partial _yn_0 = \partial _x^2y_0\left( {D^{{\mathrm{eff}}}\partial _yn_0 + 2D^{{\mathrm{swim}}}\partial _yQ_0} \right) + 4D^{{\mathrm{swim}}}\partial _x\partial _yP_0 = \\ \partial _x^2y_0\left( {D^{{\mathrm{eff}}}\partial _yn_0 + 2D^{{\mathrm{swim}}}\partial _yQ_0} \right) - 8D^{{\mathrm{swim}}}\left( {\partial _x^2y_0} \right)\partial _yQ_0 = - 4 D^{{\mathrm{swim}}}\partial _x^2y_0\partial _yn_0\end{array}$$

Thus, canceling ∂_*y*_*n*_0_, from the above equation immediately follows the condition for the front evolution

18 $$\partial _ty_0 = - 4D^{{\mathrm{swim}}}\partial _x^2y_0$$

Thus, the front is always unstable, and the growth rate is simply 4*D*^swim^. The analysis also can be done rigorously using the solvability condition^27^.

### Numerical methods

Multiphysics computational engineering platform ANSYS was used in the calculation of flow past a rotating particle. The CFX high-performance computational fluid dynamics software tool was applied. To reduce the number of mesh points, periodic boundary condition in the azimuthal direction and an open boundary condition on the exterior of the integration domain were applied.

To solve Eqs. (5) and (6), a massive parallel algorithm was implemented on the graphical processing units, and a semi-implicit code based on the fast Fourier transformation (FFT) was used to minimize the lattice artifacts. The integration was performed in a double periodic square domain of size *L* = 200, 1024 × 1024 FFT harmonics were used. The following scaling of variables in Eqs. (5) and (6) were employed: coordinates *r* are normalized by the length of a bacterium, *l* = 5 μm, time *t* by *t*_0_ = *l*^2^/*D*^swim^ ≈ 0.025 s, concentration *n* and the nematic order parameter **Q** are normalized by the critical concentration *n*_c_. The two remaining dimensionless parameters were set to unity: $\xi l^2{\mathrm{/}}D^{{\mathrm{swim}}} = n_c^2\mu l^2{\mathrm{/}}D^{{\mathrm{swim}}} = 1$. Furthermore, we set *D*^eff^/*D*^swim^ = 1.05.

### Data availability

All data generated and/or analyzed during this study are available from the corresponding author on reasonable request.

## Electronic supplementary material

Description of Additional Supplementary Files(PDF 53 kb)

Supplementary Movie 1

Supplementary Movie 2

Supplementary Movie 3

Supplementary Movie 4

Supplementary Movie 5
