# Supplementary material for: Instability of expanding bacterial droplets
Source: Nat Commun. 2018 Apr 3;9:1322. doi: 10.1038/s41467-018-03758-z (PMC5883006; doi:10.1038/s41467-018-03758-z)
Supplement: Supplementary file 1 — Description of Additional Supplementary Files(PDF 53 kb) [file 41467_2018_3758_MOESM1_ESM.pdf]

Description of Additional Supplementary Files

File Name: Supplementary Movie 1
Description: Unstable expansion of the droplet after cessation of rotation, the rotation frequency 160 Hz

File Name: Supplementary Movie 2
Description: Unstable expansion of the droplet after cessation of rotation, the rotation frequency 400 Hz

File Name: Supplementary Movie 3
Description: Stable expansion of the droplet after cessation of rotation, the rotation frequency 40Hz

File Name: Supplementary Movie 4
Description: Sequence of OCT vertical scans of bacterial distribution after cessation of rotation

File Name: Supplementary Movie 5
Description: Results of computational modeling illustrating expansion and instability of a bacterial droplet
